# Prevalence of Dyslipidaemia and Associated Risk Factors in a Rural Population in South-Western Uganda: A Community Based Survey

**DOI:** 10.1371/journal.pone.0126166

**Published:** 2015-05-14

**Authors:** Gershim Asiki, Georgina A. V. Murphy, Kathy Baisley, Rebecca N. Nsubuga, Alex Karabarinde, Robert Newton, Janet Seeley, Elizabeth H. Young, Anatoli Kamali, Manjinder S. Sandhu

**Affiliations:** 1 Medical Research Council/Uganda Virus Research Institute (MRC/UVRI), Uganda Research Unit on AIDS, Entebbe, Uganda; 2 Centre for Tropical Medicine, Nuffield Department of Medicine, University of Oxford, Oxford, United Kingdom; 3 London School of Hygiene and Tropical Medicine, London, United Kingdom; 4 Department of Public Health & Primary Care, University of Cambridge, Cambridge, United Kingdom; 5 Wellcome Trust Sanger Institute, Hinxton, United Kingdom; Shanghai Institute of Hypertension, CHINA

## Abstract

**Background:**

The burden of dyslipidaemia is rising in many low income countries. However, there are few data on the prevalence of, or risk factors for, dyslipidaemia in Africa.

**Methods:**

In 2011, we used the WHO Stepwise approach to collect cardiovascular risk data within a general population cohort in rural south-western Uganda. Dyslipidaemia was defined by high total cholesterol (TC) ≥ 5.2mmol/L or low high density lipoprotein cholesterol (HDL-C) <1 mmol/L in men, and <1.3 mmol/L in women. Logistic regression was used to explore correlates of dyslipidaemia.

**Results:**

Low HDL-C prevalence was 71.3% and high TC was 6.0%. In multivariate analysis, factors independently associated with low HDL-C among both men and women were: decreasing age, tribe (prevalence highest among Rwandese tribe), lower education, alcohol consumption (comparing current drinkers to never drinkers: men adjusted (a)OR=0.44, 95%CI=0.35-0.55; women aOR=0.51, 95%CI=0.41-0.64), consuming <5 servings of fruit/vegetable per day, daily vigorous physical activity (comparing those with none vs those with 5 days a week: men aOR=0.83 95%CI=0.67-1.02; women aOR=0.76, 95%CI=0.55-0.99), blood pressure (comparing those with hypertension to those with normal blood pressure: men aOR=0.57, 95%CI=0.43-0.75; women aOR=0.69, 95%CI=0.52-0.93) and HIV infection (HIV infected without ART vs. HIV negative: men aOR=2.45, 95%CI=1.53-3.94; women aOR=1.88, 95%CI=1.19-2.97). The odds of low HDL-C was also higher among men with high BMI or HbA1c ≤6%, and women who were single or with abdominal obesity. Among both men and women, high TC was independently associated with increasing age, non-Rwandese tribe, high waist circumference (men aOR=5.70, 95%CI=1.97-16.49; women aOR=1.58, 95%CI=1.10-2.28), hypertension (men aOR=3.49, 95%CI=1.74-7.00; women aOR=1.47, 95%CI=0.96-2.23) and HbA1c >6% (men aOR=3.00, 95%CI=1.37-6.59; women aOR=2.74, 95%CI=1.77-4.27). The odds of high TC was also higher among married men, and women with higher education or high BMI.

**Conclusion:**

Low HDL-C prevalence in this relatively young rural population is high whereas high TC prevalence is low. The consequences of dyslipidaemia in African populations remain unclear and prospective follow-up is required.

## Introduction

Dyslipidaemia is a major modifiable risk factor for cardiovascular disease accounting for an estimated 4 million deaths per year worldwide. [1] The INTERHEART study showed that dyslipidaemia is the leading population level risk factor for ischaemic heart disease in Africa. [2] Age-standardized mortality from cardiovascular diseases for countries in sub-Saharan Africa, including Uganda, is estimated to be at least three-fold higher than in several European countries, in part because of inadequate access to preventive interventions and treatment.[3] However, these estimates may not be accurate as they are based on limited data. Furthermore, definitions of dyslipidaemia are derived primarily from studies conducted in western populations and it is unclear if these definitions apply to African populations. Studies on dyslipidaemia in some parts of rural Africa have generally shown a low but rising prevalence. [4,5] However, there are major differences across the continent regarding ethnicities and dietary practices that may modify risk profiles across various populations.

In 2002, an estimated 31,700 deaths in Uganda were due to cardiovascular disease and this figure is projected to rise.[6] In our own study population in south-west Uganda, verbal autopsies conducted in the early 1990s and in 2008 revealed an increasing contribution of cardiovascular disease deaths among adults in this rural community. [7,8] It is not known what proportion of these deaths is attributable to dyslipidaemia. A few studies of dyslipidaemia in Uganda have shown a high prevalence among urban city residents, among patients with diabetes and HIV patients receiving highly active antiretroviral therapy (HAART). [9–11] Most population based studies use total cholesterol (TC) to define dyslipidaemia because it is a good surrogate marker for Low density lipoprotein cholesterol (LDL-C). However, low levels of high density lipoprotein cholesterol (HDL-C) have also been consistently shown by epidemiological studies as an independent risk factor for coronary heart disease.[12–14] We undertook a survey in rural Uganda and compared risk factors for dyslipidaemia using high total cholesterol and low HDL-C respectively. It is estimated that targeting risk factors for cardiovascular diseases at the population level could avert more than 50% of the associated deaths and disability by a combination of simple, low cost, national and individual efforts. [15]

## Methods

### Study design and study population

A cross sectional study was conducted from January to November 2011 in rural south-western Uganda as part of the annual surveys of the General Population Cohort (GPC). [16] In brief, the GPC is a community-based open cohort study with approximately 22,000 residents of 25 neighbouring villages within one half of a sub-county. The population is scattered across the county-side in villages defined by administrative boundaries with a few concentrated in small trading centres. Annual census and medical surveys have been conducted in this population since 1989. During survey round 22 in 2011, data collection included cardiovascular risk assessment. From the census of the same study round, participants were selected based on age (≥13years) and residence (lived more than three months in study area previous to survey). The interviewers contacted consented individuals in their households. All eligible and consenting volunteers within a household were included in the survey.

### Ethics

This study was approved by the Science and Ethics Committee of the Uganda Virus Research Institute ((GC/127/12/11/06), the Ugandan National Council for Science and Technology (HS870), and the East of England-Cambridge South (formerly Cambridgeshire 4) NHS Research Ethics Committee UK (11/H0305/5). A written informed consent was obtained from participants before study procedures and a copy was filed as approved by ethics committees.

### Measurements

Socio-demographic data were gathered by interviewers who administered questionnaires to a household head or any adult representative. The WHO STEPwise Approach to Surveillance questionnaire was used to collect cardiovascular risk data from individuals.[17] Biophysical measurements (blood pressure, weight, height, waist and hip circumferences) and biochemical analysis (HbA1c, TC, HDL-C, and triglycerides (TG)) were performed using standardised procedures described elsewhere. [18] Blood pressure was measured on the right arm using appropriate cuff sizes (regular arm cuff size if arm circumference was 24-32cm, large arm cuff if arm circumference was 33-41cm, thigh cuff if over 41cm and if under 24cm paediatric cuff size was used) in a sitting position, three times within resting intervals of 5 minutes, using a digital sphygmomanometer (the Omron M4-I). The mean of the second and third reading was taken for analysis. Body weight was measured using the Seca 761 mechanical scales and body height was measured using a portable Leicester stadiometer to the nearest 1 kg and 0.1 cm respectively, with participants wearing light clothing and no shoes. Waist and hip circumferences were measured twice over one layer of light clothing using a non-stretchable Seca 201 Ergonomic Circumference Measuring Tape to the nearest 0.1 cm. A third measurement was taken if the first two measurements differed by more than 3cm. Waist and hip circumferences were taken as the mean of two (or three where applicable) measurements. The weight scales were calibrated using standard weights and the height scale and measuring tape were calibrated using a standard one metre metallic rod every week. LDL-C was estimated by modified Friedwald formula: [19] LDL-C = TC-(HDL-C +TGX0.16) mmol/L.

### Definitions

Socioeconomic status was measured using an asset index, created by using principal component analysis to combine data on household possessions. Low physical activity was defined as achieving < 5 days a week of any combination of walking, moderate- or vigorous-intensity activities and <600 minutes of physical activity per week. [20] Insufficient fruit and vegetable consumption was defined as <5 servings of fruit or vegetables a day. Past, current and frequency of smoking and alcohol intake were assessed by self-report. Raised blood pressure (BP) was defined BP ≥ 140-systolic (SBP) and/or ≥90 mmHg-diastolic (DBP) or being on drug therapy and pre-hypertension as (SBP 120–139 or DBP 80–90 mmHg).[21]. Abdominal obesity was assessed using waist-hip ratio (WHR) cut-offs of 0.95 for men and 0.80 for women, [22] or waist circumference (WC) ≥94 cm for men and ≥80 cm for women. [23]HbA1c >6% for high risk category according to the WHO expert report was used.[24] Dyslipidaemia was defined by National Cholesterol Education Program (NCEP) guidelines as; TC ≥5.2 mmol/l, or HDL-C<1 mmol/l for men and <1.3 mmol/l for women, TG ≥ 1.7 mmol/l, and LDL-C≥3.4 mmol/l.[25]

### Statistical analysis

Stata11 (Stata Corporation, College Station, USA) was used for analyses. Baseline characteristics were tabulated stratified by sex. The prevalence of abnormal serum lipid levels was calculated, stratified by age and sex; 95% confidence intervals (CI) were obtained using Taylor-linearised variance estimators to account for clustering within households. In exploring the correlates for dyslipidaemia we defined dyslipidaemia as the prevalence of TC ≥ 5.2 mmol/l, or HDL-C<1 mmol/l for men or <1.3 mmol/l for women as the dependent variables separately. We estimated odds ratios (OR) and 95%CI for associations with dyslipidaemia using random effects logistic regression to account for correlation within households. Potential correlates of dyslipidaemia were examined using a conceptual framework with four levels: socio-demographic factors, lifestyle, diet and -physical activity, anthropometric factors, and clinical factors. Data on HIV treatment was extracted from our own clinic database and subjects on drug treatment for dyslipidaemia, hypertension, and diabetes were excluded. Analyses were stratified by sex because we believed a priori that some associations might differ between men and women. Age, as a potential confounder, was included in all models *a priori*. First, socio-demographic factors for which age-adjusted associations with dyslipidaemia were significant at p<0.10 were included in a multivariable model; those remaining independently associated at p<0.10 were retained in a core model. Diet and physical activity factors were added to this core socio-demographic model one by one. Factors that were associated with dyslipidaemia at p<0.10, after adjusting for socio-demographic factors, were included in a multivariable model and retained if they remained significant at p<0.10. Associations of dyslipidaemia with anthropometric and then clinical factors were determined in a similar way. The final model excluded factors one at a time until all remaining factors were significant at p<0.10.

## Results

### Characteristics of study participants

Of 8,309 individuals contacted for the study, 7,809 (94%) participated in the survey, 68 missed lipid data and were excluded, leaving 7,741 (93.2%) participants (men = 3,383, women = 4,358) in the analysis. Table 1 is a summary of participants’ characteristics stratified by sex. About half of participants (49.4%) were below the age of 30 years, 43.7% were married, 10.3% had education below primary and 44.1% had incomplete primary education, 58.7% were Catholics, 16.6% Protestants and 23.5% Muslims and 75.2% of the population was the indigenous Buganda tribe. Only 8.2% reported that they were currently smoking and 27.5% were currently consuming alcohol. Fruit and vegetable consumption ≥5 servings per day were reported by 23.4% of participants. Vegetable oil was the most common type of fat reported to have been used for cooking and 22.8% of the study participants were underweight (BMI <18kg/m^2^).

**Table 1:** Description of study participants in survey.

	Men	Women	Overall
	(N = 3383)	(N = 4358)	(N = 7741)
**SOCIO-DEMOGRAPHIC/ECONOMIC FACTORS** ^1^			
**Age (years)**			
<30	1812 (53.6%)	2013 (46.2%)	3825 (49.4%)
30–39	490 (14.5%)	811 (18.6%)	1301 (16.8%)
40–49	436 (12.9%)	601 (13.8%)	1037 (13.4%)
50+	645 (19.1%)	933 (21.4%)	1578 (20.4%)
Marital status			
Married	1406 (41.6%)	1972 (45.3%)	3378 (43.7%)
Divorced/separated/widowed	359 (10.6%)	1114 (25.6%)	1473 (19.0%)
Single	1616 (47.8%)	1269 (29.1%)	2885 (37.3%)
Education level			
Less than primary	247 (7.3%)	552 (12.7%)	799 (10.3%)
Incomplete primary	1625 (48.0%)	1791 (41.1%)	3416 (44.1%)
Primary	638 (18.9%)	866 (19.9%)	1504 (19.4%)
Junior/secondary	716 (21.2%)	975 (22.4%)	1691 (21.8%)
Above secondary	157 (4.6%)	174 (4.0%)	331 (4.3%)
Religion			
Catholic	1978 (59.8%)	2473 (57.8%)	4451 (58.7%)
Protestant	549 (16.6%)	733 (17.1%)	1282 (16.9%)
Muslim	778 (23.5%)	1073 (25.0%)	1851 (24.4%)
Other	2 (0.1%)	3 (0.1%)	5 (0.1%)
Tribe			
Muganda	2451 (74.3%)	3250 (75.9%)	5701 (75.2%)
Rwandese	498 (15.1%)	627 (14.6%)	1125 (14.8%)
Other	351 (10.6%)	402 (9.4%)	753 (9.9%)
SES score tertile^2^			
Low	823 (27.6%)	967 (25.0%)	1790 (26.2%)
Middle	1098 (36.8%)	1449 (37.5%)	2547 (37.2%)
High	1062 (35.6%)	1446 (37.4%)	2508 (36.6%)
LIFESTYLE, DIET AND EXERCISE^3^			
Smoking			
Current smoker	545 (16.1%)	89 (2.0%)	634 (8.2%)
Ex-daily smoker	166 (4.9%)	26 (0.6%)	192 (2.5%)
Never smoked daily/never smoked	2672 (79.0%)	4243 (97.4%)	6915 (89.3%)
Ever daily smoker			
Yes	673 (19.9%)	94 (2.2%)	767 (9.9%)
Alcohol consumption			
Current drinker	1109 (32.8%)	1020 (23.4%)	2129 (27.5%)
No drinking in past year	239 (7.1%)	383 (8.8%)	622 (8.0%)
Never drinker	2035 (60.2%)	2955 (67.8%)	4990 (64.5%)
5+ servings fruit/veg per day			
Yes	837 (24.8%)	978 (22.5%)	1815 (23.4%)
Type of oil used in cooking			
Vegetable oil	2814 (83.4%)	3524 (80.9%)	6338 (82.0%)
Animal fat	236 (7.0%)	348 (8.0%)	584 (7.6%)
No particular type	218 (6.5%)	309 (7.1%)	527 (6.8%)
None	105 (3.1%)	174 (4.0%)	279 (3.6%)
Vigorous physical activity/week^4^			
None	1330 (39.3%)	2182 (50.1%)	3512 (45.4%)
1–2 days	343 (10.1%)	411 (9.4%)	754 (9.7%)
3–4 days	298 (8.8%)	295 (6.8%)	593 (7.7%)
5+ days	1411 (41.7%)	1470 (33.7%)	2881 (37.2%)
Level of physical activity^5^			
High	2386 (70.5%)	2369 (54.4%)	4755 (61.4%)
Moderate	372 (11.0%)	527 (12.1%)	899 (11.6%)
Low	624 (18.5%)	1462 (33.5%)	2086 (27.0%)
ANTHROPOMETRIC INDICATORS^6^			
BMI (kg/m^2^)			
Underweight (<18.5)	1017 (30.3%)	667 (16.5%)	1684 (22.8%)
Normal (18.5–24.9)	2164 (64.5%)	2644 (65.4%)	4808 (65.0%)
Overweight (25.0–29.9)	157 (4.7%)	568 (14.0%)	725 (9.8%)
vObese (≥ 30)	18 (0.5%)	165 (4.1%)	183 (2.5%)
Abnormal Waist circumference			
≥ 94 cm (men) / ≥ 80 cm(women)	49 (1.5%)	1256 (31.0%)	1305 (16.9%)
Abnormal Waist/hips ratio			
>0.95 (men)/>0.85 (women)	122 (3.6%)	2895 (71.5%)	3017 (40.7%)
CLINICAL INDICATORS^7^			
Blood pressure group^8^			
(Systolic/Diastolic (mmHg))			
Pre-hypertension (120–139/80–89)	1399 (42.6%)	1545 (37.4%)	2944 (39.7%)
Stage I hypertension			
(140–159/90–99)	343 (10.4%)	326 (7.9%)	669 (9.0%)
Stage II hypertension			
(≥160/100)	116 (3.5%)	165 (4.0%)	281 (3.8%)
HIV serostatus			
Positive not on HAART	140 (4.1%)	243 (5.6%)	383 (5.0%)
Positive on HAART	64 (2.0%)	144 (3.3%)	208 (2.7%)
HbA1c (%)			
High (>6.0)	176 (5.2%)	253 (5.8%)	429 (5.6%)

^1^ Missing marital status for 2 male and 3 females. Missing data on tribe for 83 males and 79 females. Missing data on religion for 78 males and 76 females.

^2^ SES score computed from asset index based on household ownership of items in Round 19. Missing data for 400 males and 496 females.

^3^ Missing data on fruit/veg servings for 13 males and 9 females. Missing data on matooke and starch intake for 4 males and 6 females. Missing data on salt intake for 1135 males and 321 females. Missing data on oil for 10 males and 3 females. Missing data on physical activity for 1 male.

^4^Defined as spending at least 10 minutes continuously in vigorous-intensity activity per day; from WHO STEPS Chronic Disease Surveillance.

^5^Overall level of physical activity based on total minutes spent in vigorous and moderate activity each week.

^6^ Excludes 278 female who were pregnant at time of survey. Missing BMI for 27 males and 36 females. Missing waist/hip ratio for 20 males and 29 females. Missing waist circumference for 19 males and 26 females.

^7^Missing blood pressure for 8 males and 9 females. Missing HIV status for 3 males and 3 females.

^8^ Excludes 303 participants who report that they have taken blood pressure medication in the past two weeks.

Some characteristics differed between men and women. Women were more likely than men to be overweight (14.0% vs. 4.7%) or obese (4.1% vs. 0.5%); with large WC (31.0% vs. 1.5%) or high WHR (71.5% vs. 3.6%); with low physical activity (33.5% vs. 18.5%); and with HIV infection (8.9% vs. 6.0%). More men than women reported daily smoking (19.9% vs.2.2%), current alcohol consumption (32.8% vs. 23.4%) and were underweight (30.3% vs. 16.5%).

### Prevalence of dyslipidaemia and other abnormal lipid levels

The highest prevalence was for low HDL-C [71.3%, 95%CI (70.2–72.3)] followed by high TC [6.0% (5.5–6.6)], high LDL-C [5.2% (4.7–5.7)] and high TG [5.0% (4.6–5.5)]. Prevalence of dyslipidaemia was higher among women compared to men; Low HDL-C (78.9% vs. 61.4%), TC (8.1 vs. 3.3), high LDC-C (7.1% vs. 2.7%) and TG (5.9% vs. 3.9%). Among those with low HDL-C, 82.5% of women and 85.9% of men had normal TG levels and similarly, 95.7% of women and 99.2% of men with low HDL-C had normal TC.

**Fig 1:**
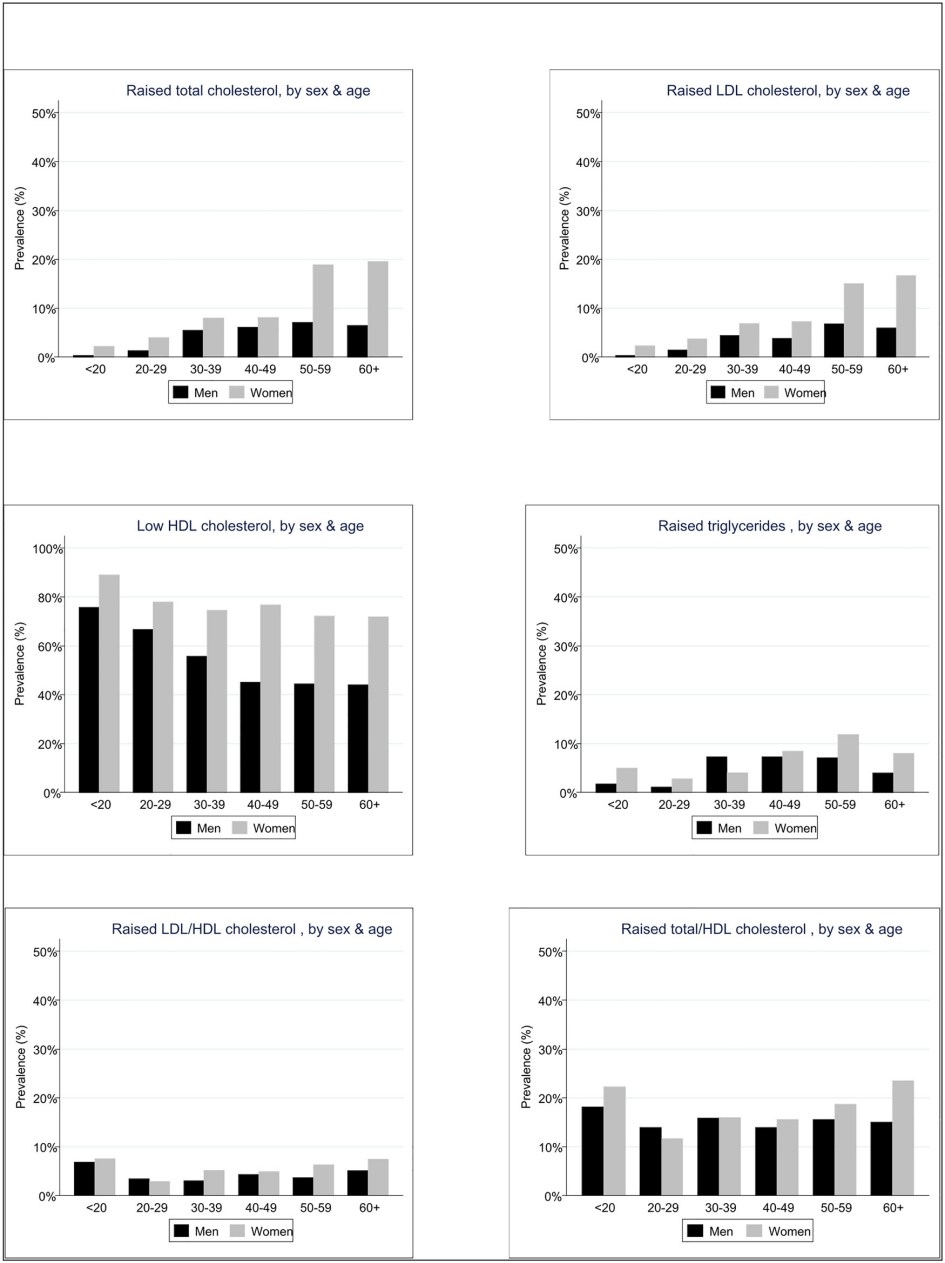
Prevalence of abnormal lipid levels by age and sex.

As shown in Fig 1, prevalence of high TC, high LDL-C, high TG, and high LDL-C/HDL-C increased with age. Low HDL prevalence decreased marginally with age.

### Factors associated with dyslipidaemia

Table 2, shows factors associated with of low HDL-C and TC respectively, after adjusting for age and stratifying by sex. In both men and women, low HDL-C was associated with marital status (prevalence highest among single), tribe (prevalence highest among Rwandese), lower levels of education, lower socioeconomic status, alcohol consumption (prevalence lowest among current drinkers), vigorous physical activity (prevalence highest among those engaging in 5 days of vigorous activity) and HIV status (prevalence highest among HIV infected not on antiretroviral therapy (ART), and lowest in those HIV infected on ART). Among men, eating fewer than 5 servings of fruit/vegetables daily was associated with increased odds of low HDL, but among women, the association was reversed. Among men only, there was some evidence of decreasing odds of low HDL-C among those with hypertension compared with normal blood pressure, and those with HbA1c >6%. Among women only, increased amount of salt added in cooking, waist circumference >80cm and high waist to hip ratio were associated with higher odds of low HDL-C.

**Table 2:** Association of factors with low HDL (<1 mmol/L in men, or <1.3 mmol/L in women), and high total cholesterol (≥5.2 mmol/L), stratified by sex.

	Low HDL				High total Cholesterol			
	Men (N = 3268) ^1^		Women (N = 4118) ^1^		Men (N = 3268) ^1^		Women (N = 4118) ^1^	
Characteristics	With low	Age-adjusted OR	With low	Age-adjusted OR	With high	Age-adjusted OR	with high	Age-adjusted OR
	HDL, n (%)	(95% CI)	HDL, n (%)	(95% CI)	TC, n(%)	(95% CI)	TC, n(%)	(95% CI)
**SOCIO-DEMOGRAPHIC FACTORS**								
**Age (years)**								
<30	1305 (73.1%)		1674 (84.6%)		12 (0.7%)		57 (2.9%)	
30–39	266 (55.6%)		589 (74.7%)		27 (5.6%)		60 (7.6%)	
40–49	191 (46.0%)		422 (76.6%)		25 (6.0%)		38 (6.9%)	
50+	257 (43.6%)		581 (72.5%)		40 (6.8%)		138 (17.2%)	
**Marital status**		P = 0.05		P<0.001		P = 0.05		P<0.001
Single (never married)	1170 (73.4%)	1	1079 (86.6%)	1	11 (0.7%)	1	27 (2.2%)	1
Married	680 (51.2%)	0.70 (0.52–0.94)	1426 (76.2%)	0.62 (0.48–0.80)	80 (6.0%)	2.30 (0.86–6.15)	160 (8.5%)	2.19 (1.32–3.61)
Divorced/sep/widowed	168 (48.7%)	0.70 (0.48–1.01)	758 (76.0%)	0.74 (0.54–1.02)	13 (3.8%)	1.25 (0.40–3.87)	106 (10.6%)	1.46 (0.83–2.57)
**Tribe**		P<0.001		P<0.001		P = 0.002		P<0.001
Muganda	1427 (60.2%)	1	2421 (78.8%)	1	80 (3.4%)	1	238 (7.7%)	1
Rwandese	330 (68.3%)	1.88 (1.44–2.45)	502 (84.9%)	1.66 (1.27–2.17)	6 (1.2%)	0.27 (0.11–0.65)	29 (4.9%)	0.50 (0.33–0.75)
Other	214 (64.3%)	1.51 (1.12–2.04)	286 (75.7%)	0.87 (0.66–1.16)	12 (3.6%)	0.87 (0.45–1.67)	23 (6.1%)	0.68 (0.43–1.08)
**Education level**		P<0.001		P<0.001		P<0.001		P<0.001
Secondary or above	470 (56.1%)	1	853 (77.3%)	1	42 (5.0%)	1	79 (7.2%)	1
Primary	375 (60.6%)	1.39 (1.07–1.79)	651 (78.9%)	1.26 (0.98–1.60)	19 (3.1%)	0.49 (0.26–0.91)	59 (7.2%)	0.76 (0.52–1.10)
None/incomplete primary	1174 (64.8%)	1.67 (1.36–2.05)	1762 (80.5%)	1.62 (1.31–2.00)	43 (2.4%)	0.39 (0.23–0.64)	155 (7.1%)	0.47 (0.34–0.65)
**SES score tertile**		P = 0.02		P = 0.04		P<0.001		P<0.001
Low	509 (63.8%)	1	718 (79.6%)	1	19 (2.4%)	1	57 (6.3%)	1
Middle	672 (63.6%)	0.83 (0.65–1.06)	1126 (81.3%)	1.06 (0.84–1.35)	21 (2.0%)	1.00 (0.53–1.89)	89 (6.4%)	1.17 (0.82–1.67)
High	617 (60.6%)	0.71 (0.55–0.91)	1064 (78.1%)	0.81 (0.64–1.03)	53 (5.2%)	2.80 (1.60–4.91)	113 (8.3%)	1.86 (1.31–2.65)
**SMOKING, DRINKING AND DIET**								
**Current smoker**		P = 0.77		P = 0.84		P = 0.75		P = 0.99
No	1764 (64.2%)	1	3205 (79.4%)	1	75 (2.7%)	1	283 (7.0%)	1
Yes	255 (48.9%)	0.96 (0.75–1.23)	61 (74.4%)	0.94 (0.54–1.66)	29 (5.6%)	0.92 (0.56–1.52)	10 (12.2%)	0.99 (0.49–2.04)
**Alcohol consumption**		P<0.001		P<0.001		P = 0.11		P = 0.78
Never drinker	1394 (70.2%)	1	2327 (82.3%)	1	33 (1.7%)	1	175 (6.2%)	1
No drinking in past year	145 (65.3%)	1.27 (0.89–1.82)	270 (80.8%)	1.07 (0.77–1.48)	8 (3.6%)	1.07 (0.44–2.57)	30 (9.0%)	0.86 (0.56–1.33)
Current drinker	480 (45.3%)	0.50 (0.40–0.62)	669 (69.9%)	0.57 (0.46–0.69)	63 (5.9%)	1.66 (0.99–2.79)	88 (9.2%)	0.95 (0.71–1.28)
**≥5 serving fruit/veg/day**		P = 0.05		P = 0.01		P = 0.47		P = 0.40
Yes	493 (60.5%)	1	765 (82.3%)	1	26 (3.2%)	1	59 (6.4%)	1
No	1518 (62.2%)	1.22 (1.00–1.48)	2494 (78.4%)	0.77 (0.63–0.95)	77 (3.2%)	0.83 (0.50–1.37)	234 (7.4%)	1.14 (0.84–1.56)
**Oil used in cooking**		P = 0.07		P = 0.57		P = 0.03		P = 0.19
Vegetable oil	1734 (63.5%)	1	2683 (79.9%)	1	78 (2.9%)	1	237 (7.1%)	1
Animal fat	127 (55.7%)	0.77 (0.55–1.07)	249 (75.9%)	0.82 (0.60–1.10)	16 (7.0%)	2.59 (1.28–5.24)	21 (6.4%)	0.80 (0.49–1.30)
No specific type	98 (48.3%)	0.70 (0.49–1.00)	210 (77.8%)	1.07 (0.76–1.50)	7 (3.4%)	0.72 (0.30–1.75)	24 (8.9%)	0.78 (0.49–1.24)
None used	56 (58.3%)	1.26 (0.76–2.08)	121 (75.6%)	0.94 (0.62–1.44)	3 (3.1%)	0.61 (0.17–2.23)	11 (6.9%)	0.56 (0.29–1.08)
**Physical activity/week**		P = 0.04		P = 0.003		P = 0.02		P = 0.31
5+ days	902 (65.7%)	1	1136 (82.5%)	1	22 (1.6%)	1	91 (6.6%)	1
1–4 days	435 (69.6%)	1.11 (0.87–1.41)	538 (79.9%)	0.70 (0.54–0.92)	14 (2.2%)	1.80 (0.86–3.75)	41 (6.1%)	1.38 (0.92–2.07)
0 days	681 (53.7%)	0.82 (0.67–1.00)	1592 (77.0%)	0.74 (0.61–0.90)	67 (5.3%)	2.05 (1.19–3.52)	161 (7.8%)	1.07 (0.81–1.42)
**Level of physical activity**		P = 0.12		P = 0.04		P = 0.02		P = 0.14
High	1498 (64.7%)	1	1823 (81.3%)	1	51 (2.2%)	1	136 (6.1%)	1
Moderate	212 (58.4%)	0.86 (0.66–1.13)	403 (79.0%)	0.88 (0.67–1.14)	14 (3.9%)	1.47 (0.75–2.89)	30 (5.9%)	0.96 (0.63–1.47)
Low	308 (52.5%)	0.80 (0.63–1.00)	1040 (76.2%)	0.79 (0.66–0.95)	38 (6.5%)	2.05 (1.23–3.42)	127 (9.3%)	1.29 (0.98–1.69)
**ANTHROPOMETRY**								
**BMI (kg/m** ^**2**^ **)**		P = 0.61				P<0.001		P<0.001
Underweight (<18.5)	632 (63.6%)	0.93 [0.76, 1.12]	537 (83.4%)	P = 0.14	17 (1.7%)	0.72 (0.39–1.31)	23 (3.6%)	0.63 (0.39–1.00)
Normal (18.5–24.9)	1284 (61.3%)	1	2055 (79.3%)	1.24 [0.96, 1.61]	65 (3.1%)	1	14 (5.5%)	1
Overweight (25.0–29.9)	81 (56.3%)	1.03 [0.68, 1.55]	404 (78.0%)	1	18 (12.5%)	4.20 (2.05–8.63)	70 (13.5%)	2.59 (1.84–3.63)
Obese (≥ 30)	8 (66.7%)	2.13 [0.50, 8.99]	120 (82.2%)	1.01 [0.78, 1.31]	3 (25.0%)	11.78 (2.03–68.35)	26 (17.8%)	3.66 (2.17–6.15)
**High waist circumference**								
**≥ 94 cm (M)/ ≥ 80 cm (F)**		P = 0.57		P = 0.008		P<0.001		P<0.001
No	1992 (62.0%)	1	2188 (80.0%)	1	91 (2.8%)	1	115 (4.2%)	1
Yes	17 (42.5%)	0.81 (0.38–1.70)	926 (80.2%)	1.31 (1.07–1.60)	12 (30.0%)	10.07 (3.60–28.16)	147 (12.7%)	2.63 (1.94–3.55)
**Waist/hips ratio**		P = 0.97		P<0.001		P = 0.36		P = 0.13
Normal	1952 (62.0%)	1	848 (76.1%)	1	96 (3.1%)	1	63 (5.7%)	1
Abdominal obesity	56 (53.8%)	1.01 (0.63–1.63)	2263 (81.6%)	1.47 (1.22–1.78)	7 (6.7%)	1.56 (0.62–3.91)	199 (7.2%)	1.27 (0.93–1.73)
**CLINICAL FACTORS**								
**Blood pressure group**		P<0.001		P = 0.17		P<0.001		P = 0.006
Normal	955 (67.5%)	1	1700 (81.4%)	1	18 (1.3%)	1	94 (4.5%)	1
Pre-hypertension	855 (61.5%)	0.84 (0.70–1.01)	1204 (78.5%)	0.89 (0.74–1.07)	46 (3.3%)	2.30 (1.29–4.13)	134 (8.7%)	1.60 (1.19–2.13)
Hypertension	204 (44.6%)	0.52 (0.40–0.68)	356 (73.0%)	0.78 (0.59–1.03)	40 (8.8%)	5.04 (2.58–9.83)	64 (13.1%)	1.45 (0.99–2.13)
**HIV infection**		P = 0.44		P = 0.68		P = 0.40		P = 0.12
No	1911 (62.2%)	1	2968 (79.4%)	1	92 (3.0%)	1	273 (7.3%)	1
Yes	106 (55.5%)	1.15 (0.80–1.65)	295 (78.7%)	1.06 (0.79–1.43)	12 (6.3%)	1.36 (0.67–2.75)	20 (5.3%)	0.69 (0.42–1.13)
**HIV infection**		P<0.001		P<0.001		P = 0.63		P = 0.10
No	1911 (62.2%)	1	2968 (79.4%)	1	92 (3.0%)	1	273 (7.3%)	1
Yes, not on ART	94 (70.7%)	2.28 (1.45–3.58)	206 (85.8%)	1.74 (1.16–2.61)	7 (5.3%)	1.20 (0.50–2.91)	9 (3.8%)	0.50 (0.25–1.01)
Yes, on ART	12 (20.7%)	0.22 (0.11–0.47)	89 (65.9%)	0.55 (0.36–0.84)	5 (8.6%)	1.67 (0.57–4.91)	11 (8.1%)	1.01 (0.52–1.97)
**HbA1c**		P<0.001		P = 0.94		P<0.001		P<0.001
≤6.0%	1940 (62.6%)	1	3093 (79.3%)	1	90 (2.9%)	1	247 (6.3%)	1
>6.0%	72 (42.3%)	0.47 (0.33–0.67)	165 (78.6%)	0.99 (0.68–1.43)	14 (8.8%)	3.35 (1.79–6.29)	46 (21.9%)	4.31 (2.92–6.37)

^1^Excludes 115 males and 240 females currently on medication for hypertension, diabetes or high cholesterol.

In contrast, among both men and women, high TC was associated with marital status (prevalence lowest among single), tribe (prevalence lowest among Rwandese), higher education and higher socioeconomic status. Increased odds of high TC was also associated with being overweight or obese, high waist circumference, hypertension and HbA1c >6%. Among men but not women, there was some evidence of increasing odds of high TC with use of animal fat for cooking and reduced amount of physical activity.

In the multivariate analysis (Table 3), the factors that remained independently associated with increased odds of low HDL-C among both men and women were: decreasing age, Rwandese tribe, lower education, and being HIV infected but not on ART. Among men and women, the odds of low HDL-C were lower among current drinkers, those who did not engage in daily vigorous physical activity, and those with hypertension. Among men, eating fewer than 5 servings fruit/vegetables daily, BMI >30 kg/m^2^ and HbA1c ≤6% were independently associated with increasing odds of low HDL-C. Among women, eating at least 5 servings fruit/vegetable daily, waist circumference ≥ 80cm, high waist to hips ratio and marital status (prevalence lowest among married) were independently associated increasing odds of with low HDL-C.

**Table 3:** Final multivariable model of factors independently associated with low HDL (<1 mmol/L in men, or <1.3 mmol/L in women), and high total cholesterol (≥5.2 mmol/L), stratified by sex^1^.

	Low HDL		High total cholesterol	
Characteristics	Men	Women	Men	Women
	Adjusted OR^2^	Adjusted OR^3^	Adjusted OR^4^	Adjusted OR^5^
FACTORS	(95% CI)	(95% CI)	(95% CI)	(95% CI)
**SOCIODEMOGRAPHIC** ^**3**^				
**Age (years)**	**P<0.001**	**P<0.001**	**P = 0.01**	**P<0.001**
<30	**1**	**1**	**1**	**1**
30–39	**0.58 (0.44–0.78)**	**0.67 (0.50–0.90)**	**4.46 (1.59–12.50)**	**2.10 (1.34–3.29)**
40–49	**0.39 (0.29–0.53)**	**0.67 (0.48–0.93)**	**3.97 (1.39–11.29)**	**2.01 (1.23–3.29)**
50+	**0.37 (0.28–0.50)**	**0.50 (0.36–0.70)**	**3.92 (1.40–11.01)**	**7.04 (4.47–11.09)**
**Marital status**	*P = 0*.*31*	**P = 0.009**	**P = 0.05**	*P = 0*.*12*
Single (never married)	*1*	**1**	**1**	*1*
Married	*0*.*79 (0*.*57–1*.*08)*	**0.66 (0.50–0.89)**	**2.53 (0.90–7.17)**	*1*.*64 (0*.*94–2*.*86)*
Divorced/sep/widowed	*0*.*76 (0*.*50–1*.*15)*	**0.81 (0.57–1.16)**	**1.30 (0.38–4.44)**	*1*.*34 (0*.*72–2*.*48)*
**Tribe**	**P<0.001**	**P<0.001**	**P = 0.03**	**P = 0.02**
Muganda	**1**	**1**	**1**	**1**
Rwandese	**1.91 (1.45–2.51)**	**1.72 (1.28–2.31)**	**0.33 (0.13–0.84)**	**0.56 (0.35–0.90)**
Other	**1.55 (1.14–2.10)**	**0.81 (0.60–1.09)**	**0.95 (0.46–1.93)**	**0.71 (0.43–1.18)**
**Education level**	**P<0.001**	**P = 0.01**	*P = 0*.*14*	**P = 0.03**
Secondary or above	**1**	**1**	*1*	**1**
Primary	**1.41 (1.08–1.84)**	**1.25 (0.96–1.64)**	*0*.*61 (0*.*32–1*.*16)*	**1.04 (0.69–1.58)**
None/incomplete primary	**1.70 (1.36–2.12)**	**1.43 (1.14–1.81)**	*0*.*61 (0*.*36–1*.*03)*	**0.68 (0.47–0.98)**
**DRINKING AND DIET**				
**Alcohol consumption**	**P<0.001**	**P<0.001**		
Never drinker	**1**	**1**		
No drinking in past year	**1.12 (0.76–1.65)**	**0.99 (0.69–1.41)**		
Current drinker	**0.44 (0.35–0.55)**	**0.51 (0.41–0.64)**		
**≥5 serving fruit/veg/day**	**P = 0.02**	**P = 0.05**		
Yes	**1**	**1**		
No	**1.28 (1.04–1.57)**	**0.80 (0.64–1.00)**		
**Physical activity/week**	**P = 0.006**	**P = 0.02**		
5+ days	**1**	**1**		
1–4 days	**1.27 (0.99–1.64)**	**0.74 (0.55–0.99)**		
None	**0.83 (0.67–1.02)**	**0.76 (0.62–0.94)**		
**ANTHROPOMETRY**				
**BMI (kg/m** ^**2**^ **)**	**P = 0.04**	*P = 0*.*65*	*P = 0*.*29*	**P = 0.009**
Underweight (<18.5)	**0.78 (0.64–0.97)**	*1*.*05 (0*.*80–1*.*37)*	*0*.*90 (0*.*48–1*.*70)*	**0.80 (0.49–1.32)**
Normal (18.5–24.9)	**1**	*1*	*1*	**1**
Overweight (25.0–29.9)	**1.30 (0.85–1.99)**	*0*.*96 (0*.*71–1*.*28)*	*2*.*12 (0*.*90–4*.*98)*	**1.76 (1.12–2.59)**
Obese (≥ 30)	**2.52 (0.55–11.65)**	*1*.*30 (0*.*80–2*.*10)*	*4*.*12 (0*.*51–33*.*35)*	**2.08 (1.17–3.68)**
**Waist circumf ≥ 94 cm**				
**(M) or ≥ 80 cm(F)**	*P = 0*.*42*	**P = 0.005**	**P<0.001**	**P = 0.01**
No	*1*	**1**	**1**	**1**
Yes	*0*.*68 (0*.*27–1*.*74)*	**1.37 (1.09–1.70)**	**5.70 (1.97–16.49)**	**1.58 (1.10–2.28)**
**Waist/hips ratio**	*P = 0*.*47*	**P = 0.02**		
Normal	*1*	**1**		
Abdominal obesity	*0*.*83 (0*.*50–1*.*38)*	**1.28 (1.04–1.57)**		
**CLINICAL FACTORS**				
**Blood pressure group**	**P<0.001**	**P = 0.02**	**P<0.001**	**P = 0.03**
Normal	**1**	**1**	**1**	**1**
Pre-hypertension	**0.89 (0.73–1.08)**	**0.79 (0.65–0.96)**	**1.84 (1.01–3.34)**	**1.55 (1.11–2.14)**
Hypertension	**0.57 (0.43–0.75)**	**0.69 (0.52–0.93)**	**3.49 (1.74–7.00)**	**1.47 (0.96–2.23)**
**HIV infection**	**P<0.001**	**P<0.001**		
Negative	**1**	**1**		
Positive not on ART	**2.45 (1.53–3.94)**	**1.88 (1.19–2.97)**		
Positive on ART	**0.13 (0.06–0.31)**	**0.53 (0.34–0.84)**		
**HbA1c**	**P<0.001**	*P = 0*.*74*	**P = 0.006**	**P<0.001**
≤6.0%	**1**	*1*	**1**	**1**
>6.0%	**0.46 (0.31–0.70)**	*1*.*07 (0*.*72–1*.*58)*	**3.00 (1.37–6.59)**	**2.74 (1.77–4.27)**

^1^Excludes 115 males and 240 females currently on medication for hypertension, diabetes or high cholesterol.

^2^Adjusted for age (a priori) & all independent predictors of low HDL in males (variables in bold): tribe, education level, alcohol consumption, eating ≥5 servings fruit/veg per day, days of vigorous activity per week, BMI, blood pressure, HIV status and HbA1c.

^3^Adjused for age (a priori) & all independent predictors of low HDL in females (variables in bold): marital status, tribe, education level, alcohol consumption, eating ≥5 servings fruit/veg per day, days of vigorous activity per week, waist circumference, waist/hips ratio, blood pressure, and HIV status.

^4^Adjusted for age group (a priori) & all independent predictors of high total cholesterol in males (variables in bold): tribe, marital status, waist circumference, blood pressure group and HbA1c.

^5^Adjusted for age group (a priori) and all independent predictors of high total cholesterol in females (variables in bold): tribe, education level, BMI, waist circumference, blood pressure group and HbA1c.

In contrast, among both men and women, the odds of high TC was higher with increasing age, non-Rwandese tribe, high blood pressure, high waist circumference, and HbA1c >6. Among men, there was some evidence of increasing odds of high TC among those who were married. Among women, there was some evidence of increasing odds of high TC with higher education level and high BMI. Although the odds of high TC were higher among married women, and among men with higher education or increased BMI, there was no evidence of a significant difference.

## Discussion

Our study has provided an opportunity to make several observations on the prevalence of, and risk factors for dyslipidaemia in a rural population in Uganda. We found a high prevalence of low HDL with close to three quarters of the population having dyslipidaemia. In contrast, high TC, high LDL-C and high TG were relatively low with less than 6% of the population having any of these forms of dyslipidaemia. A number of studies have suggested strong epidemiological evidence linking low HDL-C to adverse cardiovascular outcomes.[12,26,27] This evidence indicates that a 1% decrease in HDL—C is associated with 2–3% increase in cardiovascular risk.[26] At the population level, about 50% of the variability of serum HDL-C levels is due to genetic factors [27] and the other 50% is presumably from acquired factors including overweight and obesity, physical inactivity, cigarette smoking, very high carbohydrate intakes, type 2 diabetes and drugs such as beta-blockers, anabolic steroids and progestational agents. [28–30] In our study we established a positive association for low HDL-C with some of these factors; overweight, obesity and high HbA1c levels. [31] The association showing Rwandese tribe with higher odds of low HDL-C may be linked to genetic differences in lipid metabolism established before. [32,33] Ethnicity is also known to be associated with dyslipidaemia. [34–38]

Other factors that we found to be associated with low HDL-C were; primary or lower education, consuming < 5 servings of fruit or vegetables per day, HIV infection without ART, and current alcohol intake. These findings are in line with those from previous studies. A 20-years longitudinal study in India showed the protective effect of education on dyslipidaemia. [39] Education mediates risk of cardiovascular disease through urbanization, social gradient, unemployment, social support and cohesion, food, poverty and social exclusion, and individual health behaviours. [40] Most residents in this rural population have access to similar foods from their gardens. Those with education beyond primary school might have more knowledge on healthy dietary choices. The role of fruit and vegetables in association with cardiovascular disease risk has been well elaborated.[41,42] Less than one quarter of our study population met the minimum dietary requirement of ≥5 servings of fruit and vegetables per day, thus the low HDL-C levels. The association between dyslipidaemia and HIV infection has also been established previously.[43,44] These studies suggest that the dyslipidaemia gets worse with HIV progression, as confirmed in our study with HIV infected, ART naïve patients having higher odds of low HDL-C. This effect seems to be reversed by ART. The biological mechanism for increased risk of dyslipidaemia among HIV infected-ART naïve patients is unclear, however it has been suggested that lipid peroxidation among HIV infected individuals may be responsible for alteration of cholesterol metabolism. [44] This finding highlights the need to assess HIV infected patients for dyslipidaemia regardless of their treatment status. A meta-analysis by Rimm and colleagues showed that 30g of ethanol a day increased HDL-C by 3.99mg/dl. [45] We found lower odds of low HDL-C among current alcohol drinkers suggesting a protective effect of alcohol. However, we did not measure quantities alcohol consumed to estimate protective levels of alcohol in our study population.

Our study is one of the few to systematically examine dyslipidaemia in Uganda. Prior to this, only one population based study conducted in Kasese district in western Uganda had reported on dyslipidaemia but this did not include HDL-C as a lipid biomarker. [46] Other studies with small sample sizes were conducted in urban populations and among patients attending chronic care in hospitals. [9,10] Our study population is relatively younger (included children up to 13 years), rural and with predominantly healthy individuals. This provided us the opportunity to estimate the burden of dyslipidaemia in a rural setting across diverse age groups where services for screening non-communicable diseases are very limited.

The prevalence of low HDL-C found in our study is 7 times higher than that in the study conducted among city residents in Kampala but high TC, LDL-C and TRG in the city residents were 10 times higher than in our study sample. [9] The population based study in western Uganda also found a two-fold higher prevalence of high TC than our study. [46] These differences are attributable to variability in the study populations. Comparing with literature from other rural parts of Africa we found a similar prevalence of high TC with that found in Tanzania. [5] In contrast, rural Nigeria had much lower prevalence of high TC (3.2%), high LDL-C (0.9%), low HDL (43.1%) and highTG (1.9%). [4] However, since these studies were conducted more than a decade ago, the prevalence of dyslipidaemia may have changed. We found a high prevalence (82.5% in women and 85.9%in men) of isolated low HDL-C (defined as low HDL-C in the presence of normal TG levels).[25] Isolated low HDL-C is not unique to this population; it has been shown elsewhere to be associated with coronary heart disease mortality risk, [47,48] but not in Africa. Populations with low fat diet have been associated with a high prevalence of isolated low HDL-C. [49] Low fat intake reduces levels of HDL-C by decreasing HDL-C apolipoprotein.[50,51] Since animal fat consumption is low (7%) in our study population, isolated low HDL-C prevalence may have been over-estimated; possibly the reason why unexpected associations of low HDL-C with hypertension, HbA1c and age were observed. The consequences of isolated low HDL-C on this population are unknown but can be established through a prospective follow up.

Our study had some limitations. We measured non-fasting lipid levels and therefore could have misclassified those who had recently consumed fatty foods as dyslipidaemic. However recent literature suggests that the difference between non-fasting and fasting lipid levels is not of clinical significance. [52,53] Secondly, we applied western reference values to classify risk in this population, which may have different risk profiles. Thirdly, our questionnaire lacked some details on food quality and alcohol content.

The strengths of this study include being population-based, and having a large sample size, and wide age range, allowing stratification of risk by age and sex.

In conclusion, prevalence of dyslipidaemia, as defined by low HDL-C, is very high in this relatively young rural African population. Health services need to start planning for targeted screening for dyslipidaemia among high risk groups in the rural communities, more specifically older people in routine care, those with diabetes, and the HIV infected patients. Community health workers in Kenya were successfully trained to identify undernourished children by measuring mid-upper arm circumference by use of colour coded tapes. [54] This principle could be applied for screening high risk individuals for dyslipidaemia at the community level using waist circumference measurements. Dietary education needs to target people with low education to inform them on appropriate food choices. Future research should target the role of low HDL-C as risk factor for cardiovascular disease in populations where there is low fat consumption. Prospective follow up is needed to quantify risks of CVD associated with these abnormalities in African populations.
