# Correction: Prevalence of Dyslipidaemia and Associated Risk Factors in a Rural Population in South-Western Uganda: A Community Based Survey

**DOI:** 10.1371/journal.pone.0173133

**Published:** 2017-02-24

**Authors:** Gershim Asiki, Georgina A. V. Murphy, Kathy Baisley, Rebecca N. Nsubuga, Dermot Maher, Alex Karabarinde, Robert Newton, Janet Seeley, Elizabeth H. Young, Anatoli Kamali, Manjinder S. Sandhu

Dr. Dermot Maher should be included in the author byline. He should be listed as the fifth author, and his affiliation is: Medical Research Council/Uganda Virus Research Institute (MRC/UVRI), Uganda Research Unit on AIDS, Entebbe, Uganda. The contributions of this author are as follows: Conceived and designed the experiments, and contributed reagents/materials/analysis tools.

The correct citation is: Asiki G, Murphy GAV, Baisley K, Nsubuga RN, Maher D, Karabarinde A, et al. (2015) Prevalence of Dyslipidaemia and Associated Risk Factors in a Rural Population in South-Western Uganda: A Community Based Survey. PLoS ONE 10(5): e0126166. doi:10.1371/journal.pone.0126166
